# Utilization of Carbon Nanotubes in Manufacturing of 3D Cartilage and Bone Scaffolds

**DOI:** 10.3390/ma13184039

**Published:** 2020-09-11

**Authors:** Tomasz Szymański, Adam Aron Mieloch, Magdalena Richter, Tomasz Trzeciak, Ewa Florek, Jakub Dalibor Rybka, Michael Giersig

**Affiliations:** 1 Center for Advanced Technology, Adam Mickiewicz University in Poznań, Uniwersytetu Poznańskiego 10 Street, 61-614 Poznan, Poland; tszymanski@amu.edu.pl (T.S.); amieloch@amu.edu.pl (A.A.M.); mrichter@ump.edu.pl (M.R.); giersig@amu.edu.pl (M.G.); 2 Faculty of Chemistry, Adam Mickiewicz University in Poznań, Uniwersytetu Poznańskiego 8 Street, 61-614 Poznan, Poland; 3 Department of Orthopedics and Traumatology, Poznan University of Medical Sciences, 28 czerwca 1956r. Street No. 135/147, 61-545 Poznan, Poland; tomasz.trzeciak@ump.edu.pl; 4 Laboratory of Environmental Research, Department of Toxicology, Poznan University of Medical Sciences, Dojazd 30, 60-631 Poznan, Poland; eflorek@ump.edu.pl; 5 Department of Physics, Institute of Experimental Physics, Freie Universität, Arnimallee 14, 14195 Berlin, Germany

**Keywords:** tissue engineering, biomaterials, cartilage, bone, carbon nanotubes, scaffolds, bioprinting

## Abstract

Cartilage and bone injuries are prevalent ailments, affecting the quality of life of injured patients. Current methods of treatment are often imperfect and pose the risk of complications in the long term. Therefore, tissue engineering is a rapidly developing branch of science, which aims at discovering effective ways of replacing or repairing damaged tissues with the use of scaffolds. However, both cartilage and bone owe their exceptional mechanical properties to their complex ultrastructure, which is very difficult to reproduce artificially. To address this issue, nanotechnology was employed. One of the most promising nanomaterials in this respect is carbon nanotubes, due to their exceptional physico-chemical properties, which are similar to collagens—the main component of the extracellular matrix of these tissues. This review covers the important aspects of 3D scaffold development and sums up the existing research tackling the challenges of scaffold design. Moreover, carbon nanotubes-reinforced bone and cartilage scaffolds manufactured using the 3D bioprinting technique will be discussed as a novel tool that could facilitate the achievement of more biomimetic structures.

## 1. Introduction

In recent years, due to a high incidence of cartilage and bone injuries and subsequent risk of complications in the future (e.g., osteoarthritis) [1,2,3], a significant extension of research regarding biocompatible scaffolds engineering and vast progress in biomaterials science, in general, has been observed. Through the combination of tissue engineering techniques and advancements in the synthesis of novel materials, the understanding of biomaterials has reached a new dimension. The robust development is especially seen in the field of motor organ repair concerning bone and cartilage tissue injuries in particular. Biomaterials are viewed as the next generation treatment for these conditions, resolving many of the inherent limitations of currently existing treatment procedures [4].

Articular cartilage (AC) covers the surface of the bone in diarthrodial (synovial) joints and provides a low-friction interface for a sliding motion and also propagates the applied forces to the subchondral bone [5]. The most important drawback of an AC lesion is the fact that it does not heal spontaneously, mainly due to the small number of residual cells, limited vascular supply, and lymphatic drainage. Once injured, the lesion tends to progress and starts to deteriorate a patient’s quality of life, being the 11th most frequent cause of disability globally [6,7,8].

Bones of the skeleton provide mechanical support for joints, tendons, and ligaments; they protect vital organs from damage and they have an important metabolic role as calcium and phosphate reservoirs. Unlike cartilage, bone tissue undergoes constant renewal, and in comparison to cartilage, when the bone tissue is injured, it possesses a better regeneration potential. However, many injuries such as severe fractures exceed the natural healing ability and require surgical intervention and the use of artificial bone fillers. The lack of or unsuitable interactions between materials and tissues remains a major concern, often resulting in the lack of implant integration and its failure. In terms of synthetic materials, the most prevalent problems are early failures due to interference in the healing process, caused by issues such as bleeding, infection, and the need for debridement and antibiotic treatment. In terms of porous scaffolds, often occurring problems are a lack of cell influx to the material, cell death, or the wrong nature of the created tissue due to improper microenvironment in the scaffold [9,10]. To improve the convalescence time and the quality of life, novel biomaterials are being developed [11,12].

An ideal scaffold must possess some essential characteristics to be able to mimic the natural tissue environment and therefore to serve its role in the regenerative process. It has to be made of biocompatible, bioresorbable, and biodegradable materials which can provide proper mechanical support necessary to resist mechanical stress applied to the cartilage and bone tissue. Moreover, the ultrastructure should be arranged to deliver porosity to assure interaction between the cells themselves and between the cells and the extracellular matrix (ECM). This, in turn, should promote an adequate reorganization of injured tissue by the residual cells, while the scaffolding is resorbed by the body [13,14].

Biomolecular recognition of scaffolds by cells is essential for proper cell metabolism. This recognition can be accomplished through the usage of proper materials or their modifications. One example of this approach is the use of bioactive molecules such as chains of native extracellular matrix proteins, carbon nanotubes (CNTs), or polymers, which can interact specifically with cellular receptors [15,16]. There are a plethora of other nanomaterials investigated for tissue engineering of bone or cartilage, e.g., nanofibrillated cellulose [17], electrospun nanofibers [18], mesoporous silica [19], nanohydroxyapatite [20], and bioactive glass-ceramic nanoparticles [21], which are beyond the scope of this review.

One of the most popular materials used in scaffolds for tissue engineering which was discovered in 1991 and which has revolutionized the research field is carbon nanotubes (CNTs). They are considered to be excellent reinforcements for bio-related applications due to their remarkable structural, mechanical, electrical, and thermal properties [22,23]. Their implantation in bone or cartilage may not only improve the mechanical properties of damaged bone tissue but can also stimulate bone regeneration, as nanofibers can mimic the extracellular matrix (ECM) and allow the differentiation of human mesenchymal stem cells (hMSCs) toward osteoblasts [24,25].

The limitation with most of the conventional scaffold fabrication techniques is that they do not allow the user to create precise pores, modify its distribution, or achieve high interconnectivity with detailed geometry. 3D bioprinting has been developed to overcome most of these limitations withholding the progress of tissue engineering. This technique allows the fabrication of versatile scaffolds with complex geometry that are capable of the homogenous distribution of cells and mimic the local extracellular matrix (ECM). Besides, cells can be directly blended with bioink and therefore reside in the scaffold without the need for infiltration. There are two main approaches regarding bioprinting:

(1) the biomimicry approach, where the prepared structure is aimed to be as close to the native one as possible;
(2) the self-assembly approach, which attempts to replicate environmental and structural elements, which then promotes the assembly of a proper structure.

Therefore, bioprinting may be a revolutionary tool, able to produce complex, discrete, self-organizing units for tissue development.

This review will cover the intrinsic complexity of the native articular and bone tissue ultrastructure to depict the challenges in the bioengineering of biomimetic materials. Moreover, it will focus on structural and biological aspects of three-dimensional scaffolds reinforced with CNTs which are being researched as bone and articular cartilage replacements. Furthermore, the mechanisms by which CNTs enhance such structures will be outlined, as well as research utilizing 3D bioprinting as a useful tool to overcome the limitations of conventional scaffold fabrication techniques.

## 2. Articular Cartilage Structure and Function

Cartilage tissue is a specialized connective tissue consisting of a relatively small number of cells that are embedded in a well-organized, extracellular matrix (ECM) [26]. ECM is produced by the residual cells, the chondrocytes, and is composed of two main parts—fibrous, self-assembled elements; mainly collagens and elastin. The non-collagenous part is composed of glycoproteins and glycosamicoglycans (GAGs), such as hyaluronan and proteoglycans [27]. The dry mass is composed mainly (up to 60%) of collagens, whose unique structure defines the properties and function of the proteins themselves, as well as of the whole cartilage tissue.

Collagens are a family of fibrous proteins, whose basic structural unit consists of three polypeptide chains (*α*-chains), which can self-assemble to a closely intertwined, so-called superhelix. This twist is achieved because of the periodic occurrence of glycine, proline, and hydroxyproline amino acids which allow for tight adjacency and hydrogen bonding, respectively. These building blocks, called tropocollagens, are approximately 300 nm long, they are 1.5 nm in diameter, and they possess terminal amino acid chains. They are further arranged into long fibers, and they are stabilized due to their particular amino acid composition [28]. The fibrils’ dimensions depend mainly on the type of collagen. In the articular cartilage, collagen type II is predominant, accounting for up to 90% of the total collagen content in an adult, and it forms fibrils with a diameter of up to 80 nm [29]. Type II fibers are enhanced by other collagen types (IX and XI) [30]. Collagen type IX is attached near the surface of the collagen type II fibrils in an antiparallel fashion. It has a distinct globular protein on one end, which serves a role in interactions between various other ECM molecules. On the other hand, collagen type XI crosslinks between collagen type II fibrils and enhances the integrity of the whole structure [30,31]. Together, due to the inherent complex structure, enforced with a plethora of bonds, this multi-collagen structure forms an intricate mesh responsible for the tensile strength of the whole tissue.

Other important molecules of the articular cartilage are proteoglycans, aggrecans in particular. They are located between the collagen fibrils and act as tissue organizers, regulating collagen fibrillogenesis, and interacting with growth factors and cytokines. They serve as a backbone for GAGs attachment, which is in part responsible for the proteoglycans’ function. One of the most important GAGs is hyaluronic acid, which has exceptional water-attracting properties, forming a gel environment throughout the tissue. Hence, these non-collagenous molecules account for the elastic properties of the cartilage.

**Figure 1:**
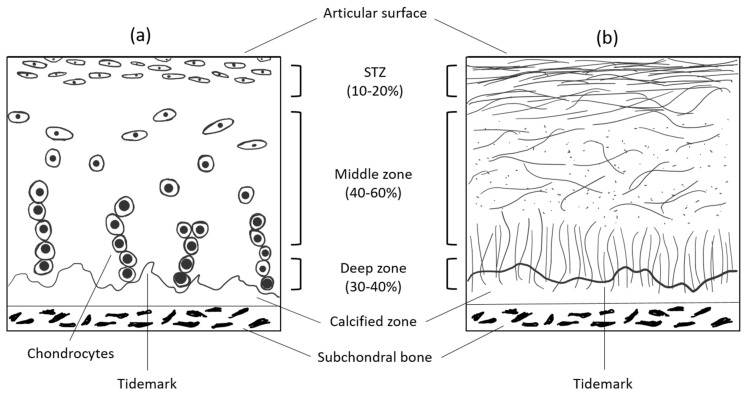
Schematic, cross-sectional diagram of healthy articular cartilage: (**a**) cellular organization in the zones of articular cartilage; (**b**) collagen fiber architecture.

This complex, multimolecular network is further organized into four distinct subcompartments, as described originally by Bennighoff [32] and confirmed by Weiss et al. [33] (Figure 1). In the first, superficial zone, which is ~200 μm thick, the fibrils are thin (32 ± 5 nm) and tend to run primarily parallel to the plane of the articular surface. Moreover, they are much more closely packed, compared to deeper layers. A greater range of fibril diameters is seen in the transitional zone II (30–60 nm) and radial zone III (40–80 nm), which are 1.5–3.5 mm thick altogether. The organization in zone II appears more random. The cells present in this zone are bigger and they produce the essential ECM components to maintain the proper microenvironment of the whole tissue. However, they represent only up to 5% of the total cartilage volume. Nevertheless, they manage to serve their role in maintaining the functionality of healthy tissue but struggle to repair it when more extensive damage occurs. In the radial zone of some joint regions, a preferred orientation of fibril bundles is vertical to the surface. The arcade-like micro-architecture of collagen responsible for this zonal appearance is anchored into the IV calcified zone.

Altogether, all of the components of articular cartilage create a very dynamic microenvironment of molecules secreted by the residual cells. This intricate ultrastructure of such a thin tissue yields exceptional tension resistance and elasticity at the same time. In general, the tensile modulus, hence the load the healthy cartilage tissue can bear, varies from 5 to 25 MPa, depending on the location, depth, and orientation within the joint surface. These well-known tensile properties demonstrate the inhomogeneous and anisotropic nature of articular cartilage [34].

## 3. Bone Structure and Function

Bone is a type of tissue with a similar composition to cartilage. Cartilage is a precursor of bone in most parts of the skeleton, gradually transforming into definitive bones during puberty and early adolescence by a process called endochondral ossification. Similar to its predecessor, the bone ECM is composed mainly of collagen fibrils; however, here type I prevails, comprising up to 90% of the organic part. This type of collagen fiber tends to have more covalent cross-linking sites than type II and therefore forms a more dense, less water-soluble, and rigid matrix, with a diameter of approximately 100 nm. This fibrous matrix is a site where the mineralization of bone occurs. Only a few types of inorganic crystals are deposited at the surface of and between fibrils, forming the basic building block of bone—mineralized collagen fibril. The vast majority of this inorganic matter consists of a plate or spindle-shaped crystals of hydroxyapatite Ca_10_(PO_4_)_6_(OH)_2._ They are very thin (2–7 nm), but polydisperse in length (15–200 nm) and width (10–80 nm). In total, they comprise about 60–70% of the bone’s dry mass, the organic part making up the remainder [35]. The water content is also much lower, between 10–20% of the total mass. In conclusion, the mineralization of the ECM, the prevalence of collagen type I, and lower hydration enhance the bone’s mechanical rigidity, which complements the tensile strength and elasticity provided by collagenous ECM.

**Figure 2:**
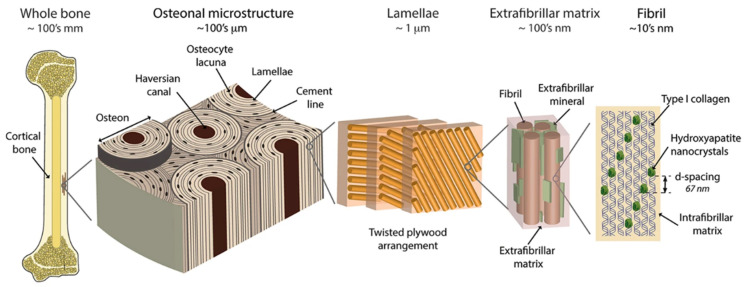
The hierarchical organization of cortical bone. On the first level, there are fibrils (~10 nm thick), composed of parallel aligned type I collagen strands, mineralized with evenly distributed hydroxyapatite crystals. Those fibrils are arranged in bundles, surrounded by extrafibrillar mineralized platelets. The bundles, arranged in the plywood-like structure form lamellae, where adjacent lamellae may have different orientation of bundles. The layers of concentrically aligned lamellae surrounding the Haversian canal forms a basic structural unit of bone—osteon (170–250 µm in diameter). Taken from [39].

The exceptional properties of bone tissue result from the intrinsic hierarchical structure in which its components are arranged (Figure 2). At the macroscopic level, two main anatomical types of bone tissue can be distinguished.

The first type is the dense cortical bone which forms the outer layers of bone and especially the shafts of the long bones. It is composed of osteons (Haversian systems), which are concentric lamellae of mineralized tissue and cells surrounding the central canal, where blood vessels are situated. These concentric fibrils are further organized into two distinct patterns: (a) dense, ordered, aligned, and parallel to the long axis of the bone, (b) disordered with randomly distributed fibers and visible porosity. These two patterns occur alternately, which yields a plywood-like structure with disordered material filling the spaces between aligned bundles of ordered fibrils [36,37,38].

The second anatomical type of human bone is called trabecular or cancellous bone (also known as spongy bone). It presents a lower density and a larger surface area than cortical bone. Trabecular bone fills the center of long bones, flat bones, and vertebrae, and consists of an interconnecting meshwork of bony trabeculae, separated by spaces filled with bone marrow [36].

The synthesis of all bone constituents, as well as maintenance of proper bone function, is carried out with residual cells of marrow origin, therefore cancellous bone has osteoinductive and osteogenic properties. Apart from the above-mentioned molecules, bone tissue is also made of non-fibrous proteins, which play an important role in many aspects of bone function and metabolism. The most notable of these proteins are osteopontin and osteocalcin, which act as a molecular Ca^2+^-mediated adhesive at the interface between mineralized fibrils. They form a large mechanical network employing the so-called sacrificial bonds and hidden lengths, which dissipate energy during tensile stretching, greatly augmenting the tensile strength of the whole bone [40]. These unique arrangements and features give the trabecular bone Young’s elastic modulus from 2 to 12 MPa and for the cortical bone as high as 12 to 18 GPa [41]. The latter is the major load-bearing part of the bone and therefore, in terms of successful bone-defect repair, the replacement material should have physical characteristics (e.g., Young’s modulus) as similar as possible to the native bone.

## 4. Current Existing and Experimental Therapies for Articular Cartilage and Bone Injuries

Nowadays, there are no established procedures for the repair of damaged cartilage. Treatments of choice for articular cartilage lesions include those which temporarily alleviate symptoms—systemic drugs like corticosteroids and painkillers, as well as injections of hyaluronan into the joint, which acts as a lubricant. Another procedure is a radical one called endoprosthesis replacement. There are three experimental approaches aiming at the permanent reconstruction of damaged tissue. The first one is an arthroscopic intervention combined with surgical access to the bone marrow, such as abrasion chondroplasty or micro fracturing, resulting in an influx of blood containing stem cells to the lumen of the joint, with the idea of the reconstruction of a new AC. The major limitation of this procedure is the complete removal of residual cartilage, even the healthy part, and subsequent immobilization of the joint for several weeks, with the recommendation of weight-bearing restriction, which affects the patient’s quality of life. The result of the healing is fibrocartilaginous tissue, which has diminished resiliency, less stiffness, and poor wear characteristics, compared to the native hyaline cartilage, resulting in poor clinical outcomes [42,43]. The second one is the surgical transplantation of the osteochondral autograft from a different region of the body or an allograft of AC from a cadaver. The major concern in this technique is patient morbidity and the outcomes vary greatly depending on age (poor in patients > 40 years), sex, and size of the lesion [44]. The last approach is autologous chondrocyte implantation (ACI). The original two-step procedure involved cartilage harvesting from a non-weight bearing surface of the affected joint, followed by cell isolation, in vitro culture, and implantation of re-differentiated cells under the periosteal cover into the defect site (1st generation ACI). Since then, the procedure has evolved and collagen membranes instead of the periosteal flap were used (2nd generation ACI), later being used as a scaffold for cultured allografted chondrocytes or autologous mesenchymal stem cells (3rd generation ACI). However, these procedures are still in the experimental or clinical trials phase with mixed outcomes and they often involve long-lasting (weeks to months) recovery after surgery. [45,46]. Since long-term follow-up studies are not yet available, it is hard to draw any conclusions as to the future application of these techniques [47,48].

In terms of bone repair, the gold standard of treatment is autologous bone grafting, despite its inherent limited availability and patient morbidity, and metal alloys bone replacement, most often titanium alloys [49]. Titanium alloys are commonly used, because of their good biocompatibility, high strength, resistance to fatigue, and high ductility, which allows for cortical bone replacement. They are used as a replacement for jaw, heel, hip, cranial bones, and spinal fusion surgeries. However, they possess two major disadvantages. Firstly, there is evidence for the release of integrating metals into the surrounding microenvironment. Its biocompatibility is provided mainly by chemical inertness due to the nanometer-thin TiO_2_ layer, which on the other hand is responsible for the lack of integration with the bone (osseointegration) often leading to implant failure. Secondly, much higher elastic modulus (120 GPa) can cause stress shielding and subsequent reduction in bone density due to the low load applied to the healthy part of the bone. One of the most successful approaches in reducing Young’s modulus of titanium implants is by preparation of porous structures with the use of 3D printing. Such structures achieved elastic modulus values as low as 36 GPa. Porous architecture could also lead to better integration if the cells would be able to infiltrate the implant (osteoconductivity). However, it requires further modification of the inert surface. Several post-treatment techniques are employed to address this issue, such as plasma spraying, ion implementation, oxidation, and alkali-heat treatment. The titanium implants are good examples of scaffolding evolution, however, their inherent limitation is difficult post-processing, possible toxicity due to metal release, and that they are not resorbable [50,51,52]. Other experimental techniques employed are composites of natural bone components, e.g., hydroxyapatite (HA) and tricalcium phosphate (TCP) ceramics or cement, with polymers (e.g., collagens, poly-lactic acid, gelatin). There is also work on using growth factors to stimulate natural bone repair processes. These composite materials combine advantages of different materials (metallic, ceramic, and polymeric materials); they allow controlled degradation with better biocompatibility and physical characteristics than the polymers alone, however, they have not yet matched the bone tissue demands in vivo [53]. Nevertheless, these procedures have their downsides with the need for surgical intervention and subsequent stable hardware fixation often being the case, because of insufficient mechanical properties of the graft, or the need to hold the graft in place.

## 5. Carbon Nanotubes

Carbon nanotubes (CNTs) are single- or multi-layered graphene sheets rolled up in the form of cylinders. The former is known as single-walled carbon nanotubes (SWCNTs), and the latter are multi-walled carbon nanotubes (MWCNTs). SWCNTs may have limited possible diameters of 0.5–2 nm. On the other hand, MWCNTs consist of multiple sheets within a tube and may have diameters of 10–150 μm, depending on the number of concentric tubes forming the structure. Their length is mostly within the range from 0.5 to 30 μm. They have a high aspect ratio, with a very wide range of possible dimensions, making them attractive for the fabrication of assembled nanoarchitectures [54,55].

### 5.1. Synthesis of CNTs

The main methods of CNT synthesis are the arc discharge method, laser ablation method, and chemical vapor deposition (CVD). These methods use an electrical breakdown of gas (arc discharge) or thermal energy to recombine the carbon precursor into carbon nanotubes. Arc discharge was the first method of obtaining CNTs, described by Ijima [56]. The major disadvantage of this method is that they are another carbon product and the catalyst (often metal such as Fe, Ni, Co) is present as an impurity. The laser ablation technique offers a higher yield of CNTs and a lower amount of metallic impurities because they tend to evaporate after laser treatment. Moreover, the diameter of the CNTs can be better controlled. However, it is not an economically viable method, due to the high cost of equipment [57]. The most common synthesis method is chemical vapor deposition (CVD), due to its lower cost, lower temperature and pressure used, and the highest yield of CNTs, in comparison to other methods. These moderate parameters offer the best control over the dimension of produced CNTs, however, they possess a relatively high amount of defects in the structure [58]. For the biological application of CNTs, the most important factor in addition to dimensionality is purity. Therefore CNTs used in biological experiments are most often synthesized by the CVD method [22,59].

### 5.2. CNT Biomimics ECM Constituents

**Figure 3:**
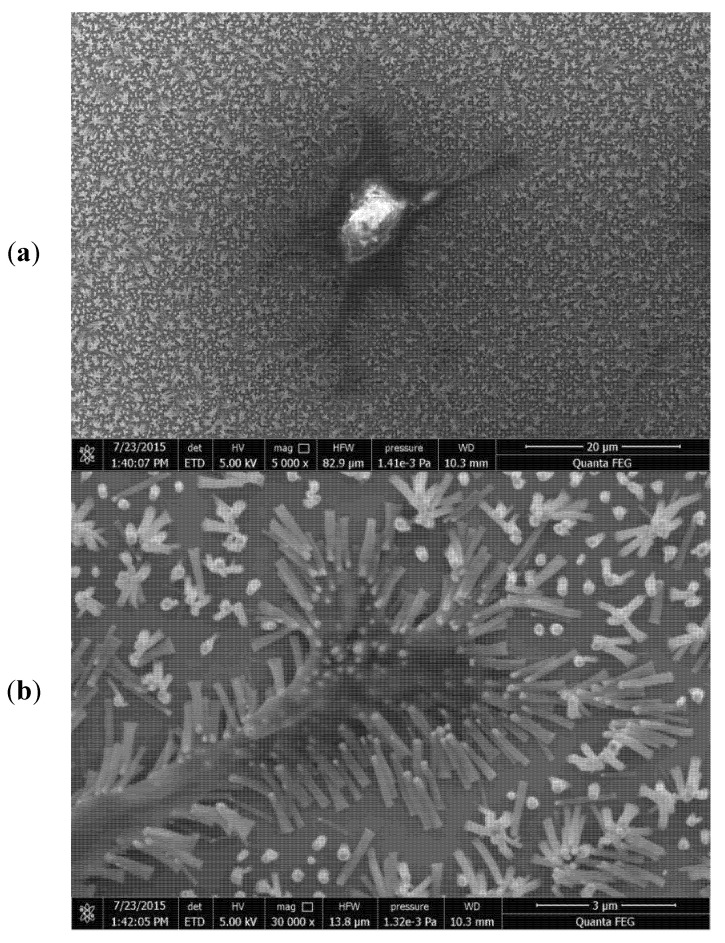
Chondrocyte was grown on perpendicularly aligned, chemical vapor deposition (CVD) synthesized multi-walled carbon nanotubes (MWCNTs), low magnification (**a**) and high magnification (**b**). Cytoplasmic extensions are visible. Note the cell adhesion to the scaffold. Phyllopodia bend the nanotubes to their purposes.

CNTs’ intrinsic graphene structure possesses inner cavities where various compounds can be internalized and/or adsorbed, such as metal ions [60], inorganic salts [61], and organic molecules [62]. This is important in terms of the scaffolding’s utility because CNTs can interact with biomolecules—nucleic acids, proteins, growth factors, collagens, adhesion molecules, and other ECM constituents. It was also found that the CNTs’ surface promotes cell adhesion [63,64]. Due to their characteristic structure, CNTs can be grown perpendicular to the plane of the surface, yielding an arrangement which can be termed the ‘‘CNT forest’’, where CNTs are densely packed, parallel to each other. Various cells including Chinese hamster ovary cells (CHO) and human chondrocytes were cultured on such a surface (Figure 3) [65]. It was found that cells were aligned along with the MWCNT bundles. Reduced clustering of cells was observed, as well as a higher proliferation rate. Moreover, 16% of the cells had an aspect ratio of 3:1 or higher. This result indicates that CNTs favor cell adhesion and can act as a platform for cell growth. Additionally, ECM is a natural scaffold, providing mechanical support for the tissue. CNTs, with their exceptional physical properties with tensile strength as high as 50 GPa, and Young’s modulus as high as 1 TPa, are a good candidate as a component of robust scaffolds.

### 5.3. CNT Toxicity

One of the fundamental topics regarding the use of CNTs in bioengineering is their safety. The effects of CNTs on various cell types have been previously extensively reviewed [66,67,68]. They are mainly in vitro studies. On the other hand, in vivo animal studies have raised concerns, especially about pulmonary toxicity, due to their asbestos-like mechanism [69]. The summary of research regarding various features of CNTs, and its impact on toxicity is presented in Table 1. As it was noted by Castranova et al., it is important to take into consideration that many of these studies may be biased because of the following respective reasons: (1) the use of doses per cell that are much higher than those possible to achieve in the natural environment, (2) CNT agglomeration, and (3) adsorption of signals emitted by assay indicator dyes [70]. Therefore, care must be taken when analyzing in vitro results. Low doses of SWCNTs, which are more feasible to be achieved with daily exposure, such as 40 μg/mouse, aspirated by mice, exhibit low cytotoxicity. Conversely, a low dose of SWCNT exposure of lung fibroblasts increases the proliferation rate and collagen production [71]. Therefore, the rapid onset of an interstitial fibrotic response to pulmonary CNT exposure may be caused by direct interactions with CNTs (matrix effect), resulting in fibroblast activation [72]. Li et al. compared the effects of MWCNTs on lungs upon administration with intratracheal instillation and inhalation. They found an inflammatory response and alveolar destruction after a single instillation of high dose MWCNTs and no inflammation with alveolar wall thickening after gradual inhalation of MWCNTs [73].

Consequently, many in vitro studies with CNTs have employed doses in the range of 10–100 μg/mL and have reported cytotoxicity. As it has been said, on a per-cell basis, such doses are orders of magnitude higher than those achieved in pulmonary exposure studies with rats or mice and may not be relevant to workplace exposure. National Institute of Occupational Safety and Health (NIOSH) researchers have determined the ratio between the CNT mass/surface area of cells, based on the lung’s epithelial surface area in humans, rats, and mice. When such low doses are used in cellular experiments, the stimulation of lung fibroblast proliferation and collagen production rather than cytotoxicity was found, which mimics the fibrogenic effects of CNTs observed after pulmonary exposure. Such low doses also reveal CNT-induced cell transformation and aneuploidy instead of cell death [74].

Another crucial factor in CNTs’ toxicity is their size. Equivocal results are published regarding toxicity and retention. For example, Takagi et al. reported the onset of mesothelioma after the intraperitoneal injection of long (28% >5 μm in length) MWCNTs [75], contrary to Muller et al., who reported no mesothelioma after the injection of 0.7 μm MWCNTs. Similarly, 81% of long MWCNTs (6 μm) were retained in the lungs after 60 days, in contrast to only 30% of short (0.7 μm) MWCNTs [76]. Similarly, Allegri et al. compared the influence of the MWCNTs’ diameter and functionalization on macrophages’ viability. With a diameter larger than 40 nm, no detrimental effects were observed, while mild cytotoxicity was found for diameters between 15 and 40 nm. However, when these MWCNTs were functionalized with either carboxyl or amine groups, the toxicity was mitigated. This phenomenon is attributed to the observed higher amount of serum proteins adsorbed on the surface of MWCNTs, which is called a protein corona. These proteins alter their surface chemistry, which eventually leads to the formation of larger aggregates, mitigating the biological effects on cells [77]. Sager et al. tested the influence of MWCNTs’ carboxyl surface modification in C57BL/6 mice after lung instillation in comparison to unmodified MWCNTs. The results showed that functionalization significantly reduced inflammation and lung pathologies. Importantly, both MWCNT suspensions were prepared with surfactant (DPCC) and mouse albumin to promote dispersion, eliminating the possible aggregation factor. Thus, the surface modification, not dispersion status, was the primary driving force behind the differential activity of the MWCTs [78].

Mutlu et al. tested whether the aggregation of SWCNTs has an impact on toxicity. They instilled intratracheally, 40 mg of unpurified, aggregated, and highly dispersed SWCNTs in 1% Pluronic F 108NF to mice. The results showed that lung inflammation was induced by aggregated SWCNTs in phosphate buffer saline (PBS), while highly dispersed SWCNTs did not cause any inflammation or fibrosis [79].

Interestingly, increased reactive oxygen species (ROS) production by macrophages after exposure to CNTs was attributed to the CNTs’ intracellular degradation. Elgrabli et al. reported two degradation mechanisms of MWCNTs with an outer diameter of 40–80 nm—a non-site specific thinning process of the walls and site-specific transversal drilling process on pre-existing defects of nanotubes. Both of these processes are mediated by the ROS produced by macrophages. Eventually, after 168 h of exposure, 51.1% of the MWCNTs’ total surface was degraded [80].

Aldieri et al. examined the role of impurities left in CNTs which were used as catalysts in the process of synthesis. The tested MWCNTs were subjected to the additional step of purification to minimize iron remnants content. Only iron-rich MWCNTs exerted a cytotoxic effect, as well as oxidative stress on murine alveolar macrophages, which proves the detrimental role of such contaminants [81].

Tutak et al. compared osteoblast behavior on SWCNTs which were dissolved freely in the media to those which were firmly attached to the surface [82]. Interestingly, after 24 h free-flowing CNTs caused cell deaths; however, the resulting release of growth factors by dying cells stimulated the remaining ones to an enhanced secretion of ECM proteins. In contrast, neither cytotoxic effects nor enhanced ECM production was observed on the cells grown on the fixed SWCNTs. In another work, Tutak et al. compared the level of adhesion of MC3T3 cells on hydrophilic CNT films with different surface roughness (60 and 100 nm) and determined that the 100 nm surface gives better adhesive properties [55].

The last essential aspect is the method used for cytotoxicity measurement. Casey et al. determined with the spectroscopic analysis that SWCNTs interact with various indicator dyes, such as Coomassie Blue, Alamar Blue^TM^, Neutral Red, MTT, and WST-1. Consequently, the employment of different methods yielded various results [83]. Similarly, Wang et al. found discrepancies in the fluorescent LDH test, depending on the wavelength used for measurement, and proposed an algorithm for compensation to obtain more accurate results [84].

These findings lead to the conclusion that nanostructuring and carefully controlling the CNTs’ sizes, doses, purity, and methods of administration are crucial in the determination of the CNTs’ toxicity. It can be stated that CNTs, while embedded, do not inflict significant toxicity, and even if it can be observed, this may have a stimulating effect on cells and, eventually, have a positive impact on the tissue as a whole. Moreover, existing research demonstrates that cells can efficiently degrade residual CNTs within a range of sizes, which may diminish the concerns of the long-term toxicity caused by CNT retention in an organism. Finally, fixed CNTs have a positive effect on cell adhesion, proliferation, and cytoskeletal development, which presents CNTs as a possible enhancement in scaffold bioengineering.

## 6. Development of Bone and Cartilage Scaffolds

ECM plays an essential role in the tissue, as it provides an optimal environment for cell attachment, proliferation, and growth, as well as proper nutrients and cytokine flow. At the same time, it possesses sufficient mechanical strength to retain the shape and structure of the tissue and plays its role in the shock absorption properties. Both cartilage and bone tissue exhibit very complex and peculiar ultrastructure which is responsible for its function. So far, the biggest challenge and the Holy Grail in tissue engineering is to obtain such a structure, which is as close as possible to the natural one in terms of these attributes. Several aspects have to be addressed in fabricating such structures.

### 6.1. Biodegradability and Biocompatibility

The ideal material for a biomimetic scaffold has to be biocompatible, because it will house the cells and it will be implemented in the body, thus should not be toxic and elicit inflammatory responses. Secondly, the material has to be biodegradable (bioresorbable)—it should decompose over time, being exchanged by the tissue produced by the hosted cells. Therefore, synthetic and natural hydrogel-forming polymers turned out to be good candidates, because they possess all of those features [85]. PVA (polyvinyl alcohol) [86], PEG (polyethylene-glycol) [87], PLGA (polylactic-co-glycolic acid) [88], PLA (polylactic acid) [89], gelatin [90], alginate [91,92], collagen [93] and chitosan [94] have all been researched as a scaffold material for tissue engineering. These materials have the properties to support cell attachment and proliferation, especially osteocytes and chondrocytes [95]. They can be enhanced by supplementing the scaffold with particular proteins such as laminin to enhance adhesion [96]. Natural polymers such as collagen have the advantage that they can trigger specific cell signaling, such as to elicit osteogenesis [97]. They also can be degraded by the enzymes in our body, and the degradation rate can be modulated by either chemical modification, or altering the manufacturing process [98].

### 6.2. Mechanical Properties

As was described earlier, cartilage and bone serve a supportive function in the body, therefore scaffold replacing the tissue should have adequate mechanical properties such as Young’s modulus. The hydrogels alone were too brittle to serve this function properly. To overcome this limitation, a lot of research has been conducted to incorporate reinforcing materials. One of the most researched reinforcing materials is PCL (poly-caprolactone). It is biocompatible, much tougher than the abovementioned polymers, and has a degradation time of two to three years. Therefore, PCL fibers are often used to prepare a composite material with hydrogel polymers. It is often electrospun or bioprinted in the form of a mesh, and eventually, the fibers are embedded in the polymer. Fibrous PCL can have an elastic modulus of 3.1 MPa, while a composite with various substances may augment its mechanical properties a few-fold [99]. Consequently, it enhances the mechanical properties of as-prepared composite materials, e.g., the composite of thick-fiber PCL (>88.5 µM), and GelMA had a stiffness of 16.1 MPa, which is similar to the native articular cartilage [100]. Another material used as a reinforcing material is cellulose nanofibers. They have a high surface area, broad chemical modification capacity, and similarity to collagen nanofibers. They have been used as a reinforcing material for alginate hydrogels for the cartilage scaffolds of human ear and sheep meniscus constructs [101]. Other research was conducted presenting good biocompatibility, cell proliferation, and extracellular matrix formation, however, Young’s modulus of as-prepared scaffolds are in the range of single to tens of kPa, which is not enough for articular cartilage and bone replacement [102,103]. CNTs, due to their mechanical and biological properties, are a perfect candidate to use as a reinforcing material, instead of plastic fibers like PCL and cellulose fibers [91,104,105].

### 6.3. Porosity

**Figure 4:**
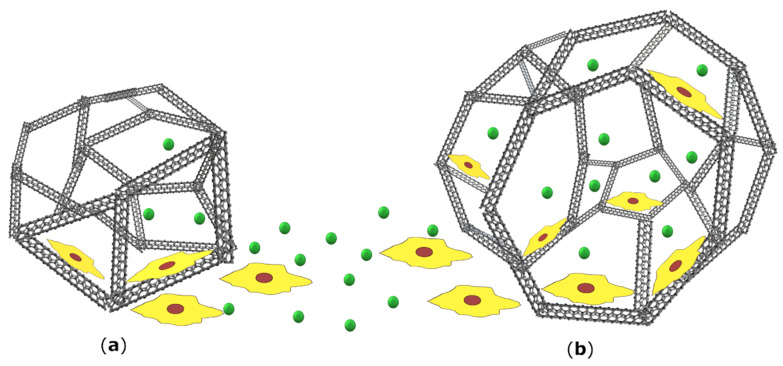
Porosity is a crucial factor in designing a scaffold. If pores are too small (**a**) there is a limited diffusion of nutrients and metabolites, as well as cell infiltration into deeper layers of the scaffold. However, such spatial constraint places cells near each other and therefore promotes proliferation, until the space becomes exhausted. On the other hand, if the pores are bigger (**b**) the flow of nutrients and cell penetration is much more efficient. Due to the low surface area, cell interactions and adhesion are exacerbated, which leads to slower cell proliferation. The figure is a schematic representation of a porous scaffold and it is not drawn to scale.

Another crucial aspect of a scaffold is the level of porosity and the size of the pores. This is important for the successful infiltration of cells, as well as diffusion of nutrients, gases, and metabolites into the deeper scaffold cavities. If they are too small, these processes are constrained. If too large, there is a limited surface area, which decreases the cell interactions and adhesion, both crucial phenomena for proper cellular function (Figure 4). On the other hand, if the material is too porous (>90%), it loses the mechanical sturdiness [13]. In terms of bone, it has been determined that the minimum pore size for bone growth is 75–100 μm with an optimal range of 100–135 μm [106]. However, it has also been reported that pores greater than 300 μm are essential for optimal vascularization [107]. Murphy and O’Brian experimented on collagen-glycosaminoglycan scaffolds with pore sizes of 96–151 μm and 85–325 μm. They found that within smaller pores, there was an initial rapid proliferation of cells; however, the process was quickly diminished. On the other hand, after a week, the cells within larger pores showed deeper penetration into the scaffold, combined with a higher number of cells overall. This was ascribed to the ease of the cellular expansion and better diffusion of nutrients [108].

## 7. Advantages of CNTs’ Usage in Scaffolds

Lalwani et al. presented a comprehensive cytocompatibility assessment of free-standing 3D constructs composed of CNTs, in comparison to the PLGA scaffold with similar porosity levels (80–90%) [109]. An average pore size, determined with the MicroCT technique, was 300–500 μm for PLGA and 100–400 μm for scaffolds prepared with MWCNTs and SWCNTs. Despite the difference in composition, the majority of tests carried out using MC3T3 cells (cytotoxicity, viability, proliferation, adhesion, infiltration) showed comparable results. Interestingly, the morphology of cells was different among the scaffolds, with the elongated, spindle-like shape and more circular shape on MWCNT and SWCNT fabricated scaffolds, respectively. It was noted that this difference may be attributed to the difference in CNTs’ dimensions. MWCNTs are thicker and longer compared to the used SWCNTs, which resulted in different surface roughness.

Joddar et al. developed an MWCNT-alginate composite by linking the alginate chains to –COOH-functionalized MWCNTs by Ca^2+^ ions. Unlike alginate alone and alginate with incorporated pristine MWCNTs, the prepared composite presented non-Newtonian pseudoplastic properties. It also presented a shear-thinning behavior characterized by decreasing viscosities with an increasing shear rate. The degradation time in the DMEM media of the scaffold was prolonged more than twofold. MWCNT addition to the amount of 1 mg/mL resulted in an increase in average pore size from 1.01 ± 0.39 μm to 1.98 ± 1.08 μm and, importantly, the pores were homogenously distributed and interconnected. This resulted in the clustering of HeLa cells in MWCNT-alginate scaffolds. This may be very promising for cartilage development since chondrocytes naturally grow in micromasses. It also resulted in a slight increase in the elastic modulus. All of these advantages are lost with increasing concentrations of MWCNTs. However, the authors declare that the level of MWCNT-alginate crosslinking is low and it can be increased by introducing more –COOH groups as well as increasing the Ca^2+^ ions’ concentration. This would resemble natural complex ECM interconnections more closely, which should result in much better mechanical properties [91]. Similar findings were published by Lau et al., who analyzed various concentration levels of chitosan and CNTs to compare the porosity in three-dimensional chitosan–CNT scaffolds. The MWCNTs used in the experiment were of precisely defined dimensions of 20–30 nm outer diameter and 10–30 μm length. This size was selected due to the chitosan properties and mechanism of synthesis since such sized CNTs would not decrease the likelihood of entanglement and therefore the need for the use of a greater concentration of CNTs. Moreover, they were significantly larger than the average diameter of the chitosan pores. Such a scaffold was prepared with the TIPS method and exhibited 92–97% porosity, with pore sizes of 100–300 μm. Interestingly, the mechanical strength of the chitosan–CNT scaffold improved with the increasing concentration of CNT; however, beyond the optimum concentration of CNT, the scaffolds became more brittle and fragile.

Natural cartilage or bone tissue is composed of a plethora of interconnected molecules, and hence structures composed of diverse molecules were investigated. For example, chitosan–MWCNTs–HA nanocomposites prepared by Chen et al. demonstrated a sharp increase in the elastic modulus by 114% (from 509.9 to 1089.1 MPa) and compressive strength by 218% from 33.2 to 105.5 MPa, by increasing the MWCNT–chitosan weight ratios from 0% to 5%. Importantly, it did not affect cell viability. These values, though not as high as for the native bone, present that cooperation between different components may vastly increase the mechanical properties [104].

Kroustalli et al. showed that osteoblasts have increased adhesion properties, as well as proliferation and differentiation potential, on even pristine MWCNTs [110]. The MWCNTs with a mean diameter of 5–20 nm and >10 um were prepared as a thin film (243.4 ± 41.3 nm) of randomly oriented networks of aggregated CNTs. The MWCNTs accelerated human mesenchymal stem cell differentiation to a higher extent (three-and-a-half fold) than a plastic tissue culture, even in the absence of additional biochemical inducing agents, measured with alkaline phosphatase (ALP) activity. The pre-treatment with anti-integrin antibodies decreased the number of adherent cells and adhesion strength at 4–60%. In conclusion, even pristine MWCNTs can adsorb proteins and therefore allow osteoblasts to adhere via the integrin-mediated pattern.

Deligianni discussed the role of scaffold dimensions concerning cell shape and its impact on cell fate [111]. It has been concluded that the cell morphology correlates with the physiological behavior of the cells, especially that the cell growth is accelerated when cell adhesion is decreased. The study presented that on flat and polished surfaces, lower phyllopodia content was correlated with an increase in cell proliferation and a more elongated and spindle-like morphology. In contrast, on microrough surfaces, the cell bodies become more cuboidal and form polygonal shapes, with an accompanying lower proliferation rate. The clear correlation between cell shape and differentiation leads to the assumption that changes in the assembly and disassembly of the actin cytoskeleton may be critical in supporting mesenchymal stem cell differentiation, especially into bone cell lineage. In conclusion, smaller MWCNTs of 30 nm in diameter promote adhesion, whereas larger (70 to 100 nm) MWCNTs elicit differentiation.

Chanine et al. cultured chondrocytes in composite mixtures of 2% agarose and two preparations of CNTs, carboxyl functionalized SWCNT–COOH, and covalent PEG-modified SWCNTs [112]. The SWCNT–COOH showed greater viability than a control scaffold, whereas the SWCNT–PEG scaffold showed inhibited proliferation. Interestingly, a higher concentration of SWCNT–PEG yielded much higher ECM production, despite the lowest cell viability. This is in concordance with the previously mentioned finding that the higher the adhesion level, the lower the proliferation rate, but the more active the metabolic state of the cell.

## 8. Methods of Scaffold Fabrication

There are several methods used for scaffold production. They are based on the formation of nanofibers from the biomaterial of choice with further attempts to organize them into desired spatial architecture. Current methods of scaffold fabrication are divided into conventional and rapid prototyping methods.

### 8.1. Conventional Techniques

Conventional techniques are defined as processes in which prepared scaffolds have continuous, uninterrupted pore structure, however, it lacks long-range channeling microarchitecture.

#### 8.1.1. Electrospinning

One of the very first methods utilized is electrospinning. Electrostatic charge is applied to the polymer in liquid form, which is extruded through a small jet, resulting in the nanofibers which are deposited on the metallic collector [113]. Conventional electrospun fibers can be aligned randomly or in the vertically aligned fashion, creating a tightly packed, 2D mesh which is similar to native ECM of some tissues, e.g., skin [114]. However, as was noted before, the cartilage and bone are complex hierarchical structures. Therefore, it was attempted to create 3D structures with higher porosity to facilitate cell infiltration. One of the methods was to electrospun the fibers directly to the liquid collector instead of the flat one. It resulted in a more porous structure, however, with limited control over spatial arrangement [115]. There are several papers where CNTs are used in a composite material with PCL [116,117] and polyurethane [118,119].

#### 8.1.2. Solvent Casting

This technique is based on the mixing of uniformly distributed salt particles in a particular solvent with a polymeric solution. After evaporation of the solvent, the matrix is submerged in water, leading to the dissolution of salt particles, leaving a porous scaffold.

#### 8.1.3. Freeze-Drying

This process is also called lyophilization. Mao et al. developed a method to synthesize a collagen-based scaffold reinforced with SWCNTs using monodisperse ice crystals [120]. The ice particles, created by spraying the ultrapure water into liquid nitrogen, were sieved with 355 μm and 425 μm sieves at −15°C. They were mixed with a collagen solution and freeze-dried after another incubation step. Finally, the scaffold was immersed in a solution of collagen-coated SWCNTs. The process resulted in homogenous, interconnected pores with a narrow size distribution of 359 ± 53 μm in diameter. After two weeks, the number of cells and GAGs synthesis was significantly greater in the SWCNT-enforced scaffolds compared to the bare collagen.

#### 8.1.4. Self-Assembly

Self-assembly is a process in which fibers arrange themselves spontaneously into fibers of the desired shape, due to intramolecular interactions. It is achieved by using particular functional motifs (e.g., amino acid sequences) [121], or chemical interactions in particular conditions (e.g., the polarity of solvent) [122]. Peptide self-assembled scaffolds have good biological activity, however, they are too brittle to carry the mechanical load as a scaffold.

#### 8.1.5. Phase Separation

Phase separation is a technique that produces 3D nanofibrous structures with nanofibers of dimensions similar to collagen fibrils of ECM (50–500 µm). This technique employs two substances that are incompatible in a particular solvent, which results in the formation of a porous structure, followed by the replacement of the solvent. The fabrication process is convenient, however, it is restricted to certain specific polymer-solvent combinations and since they have high porosity the obtained scaffold is too brittle for cartilage and bone scaffolds. [123]. The modification of this method is a thermal-induced phase separation (TIPS), which utilizes temperature difference to separate the phases.

In general, scaffolds produced by conventional methods have poorly controlled pore sizes and spatial distribution. Thus, the cellular infiltration and diffusion of nutrients are limited [124].

### 8.2. Rapid Prototyping Technologies

Rapid prototyping technologies are bottom-up approaches, which are capable of producing specially designed forms and shapes with the use of computer-aided design (CAD).

#### 8.2.1. 3D Bioprinting

3D bioprinting is a technique in which a biomaterial is precisely extruded in a layer-by-layer approach, thus giving much more control over the shape and microarchitecture of a printed scaffold. Therefore, it allows the creation of a construct that closely resembles native tissue. This is a crucial aspect, both in terms of mechanical support and for proper cellular function because, as was mentioned earlier, the local environment inside the tissue affects every aspect of cellular behavior such as metabolism, proliferation, and migration [125,126].

The biomaterial used in bioprinting is composed of a hydrogel matrix, in which living cells are uniformly suspended, and it is therefore called a bioink. An ideal bioink should have a few key features such as printability, shape stability, sufficient mechanical strength, biocompatibility, cytocompatibility, and degradability. Because it has to be bio- and cytocompatible, currently used bioinks are based mainly on natural polymers, such as alginate, agarose, gelatin, fibrin, chitosan, hyaluronate, and collagen; however, synthetic polymers, e.g., poloxamer (pluronic) or polyethylene glycol (PEG) are also exploited. Except for agarose and alginate, these materials have an inherent resemblance to the ECM. As relatively soft hydrogels, they have good printability parameters; however, they do not possess sufficient mechanical stability to resist the same continuously applied loads as a cartilage or bone replacement. Therefore, two approaches are utilized—crosslinking and augmentation with stiffer materials, generating a composite scaffold [127].

There are two types of crosslinking—physical and chemical. Physical crosslinking relies on ionic or hydrophobic interactions and hydrogen bonding. One of the most extensively used hydrogels, alginate, is physically crosslinked after printing by the addition of calcium salts (e.g., CaCl_2_). Chemical crosslinking is based on the formation of covalent bonds, and therefore as-prepared scaffolds are mechanically stronger [128]. For this type of crosslinking, various chemical modifications of polymers are used, e.g., methacrylated or diacrylated moieties of PEG, cellulose, gelatin undergoes radical polymerization after UV light irradiation [129,130,131].

Composite scaffolds are made by the usage of rigid materials, in the form of an external mesh, or fibers and particles, along with the hydrogel formulation. An example of a widely used synthetic material is polycaprolactone (PCL), due to its mechanical strength and biocompatibility. Daly et al. [132] printed a mesenchymal stem cell-laden scaffold on the pattern of vertebrae. In this study, alginate-based bioink, reinforced with PCL fibers, was utilized. This reinforcement led to a significant increase in the compressive modulus (3.867 ± 0.2187 vs. 1402 ± 157.8 kPa). This model was tested in vivo by subcutaneous implantation into mice. After 12 weeks, bioprinted vertebrae were extensively vascularized and mineralized; 24.6% ± 4.8% of the whole scaffold surface was bone tissue. Piard et al. co-printed osteon-like scaffolds with fibrin/gelatin bioink, along with a PCL supporting carrier scaffold. The application of the latter vastly increased the compressive modulus, which fell into the range of cortical bone [133]. On the other hand, Gao et al. printed a 3D scaffold made of poly(ethylene glycol)dimethacrylate (PEGDMA) in which nanoparticles of bioactive glass and hydroxyapatite were suspended [134]. The nanoparticles had a dual purpose—they reinforced the whole scaffold, increasing its compressive modulus, and hydroxyapatite was an osteogenic inducer to the mesenchymal stem cells present in the bioink.

#### 8.2.2. Laser-assisted Bioprinting

Laser-assisted bioprinting (LAB) is a nozzle-free, non-contact technique, where the laser is pulsed on a three-layer material: laser-transparent layer, laser absorbing layer, and bioink. Energy from penetrating laser is absorbed and forms bubbles, which ejects the bioink from the laser surface onto the collector stage. Kerouredan et al. used this technique to print a pattern with labeled endothelial cells directly into the bone defect in mice, which was filled with a collagen matrix. The defined local cell density of endothelial cells allowed the generation of microvascular networks [135].

#### 8.2.3. Fused Deposition Modeling

Fused deposition modeling (FDM) is a technique in which polymer filaments are inserted into a heated nozzle, melted and extruded.

#### 8.2.4. Stereolithography and Digital Light Projection

Stereolithography (SLA) and digital light projection (DLP) are nozzle-free techniques that create objects through layer-by-layer photopolymerization.

Another promising direction in 3D bioprinting is the so-called 4D bioprinting, where the concept of time is added as a fourth dimension. This means that a 3D-bioprinted scaffold can change its characteristics, spontaneously, or under various stimuli, after the printing process [136]. The stimuli-responsive materials can “remember” their shapes due to their chemical composition. For instance, thermo-responsive materials can change their shape (e.g., polyurethane [137]), or achieve sol-gel transition below a particular temperature. Fabrication of such scaffolds has many potential advantages. Wu et al. prepared injectable chitosan/silk fibroin/bioactive glass nanoparticle hydrogel, which can be injected directly into the damaged bone. In body temperature it undergoes a gelling process, yielding porous filling. It was tested in rats, and after eight weeks it restored vascularized bone tissue and mineralized collagen deposits. No cells and growth factors were used, which suggests the infiltration of the scaffold by cells and its further replacement with native bone tissue [138].

There is a limited amount of research regarding CNT usage as a supplement in bioinks. One of the most interesting works was published by Goncalves et al. [105]. A three-phased HA–CNT–PCL composite was used to print a scaffold for bone cell growth. The CNT content was up to 10%, embedded in 50% PCL, HA being the remaining part. The addition of 2% CNT yielded the best mechanical and electrical properties, with a compressive strength of ~4 MPa, which is similar to that of a trabecular bone. On the other hand, the elastic moduli were measured 44 MPa for the 2% CNT supplement. Although 0.75% CNT yielded the highest Young’s modulus (60 MPa), it was not conductive, which plays a role in regulating the physiological behavior of cells, e.g., the electrical stimulation of bone promotes osteogenesis [139], and therefore it is a desirable property for a bone scaffold. The pore sizes could be adjusted from 450 to 700 μm. Moreover, the material elicited the adhesion and spreading of osteoblast-like cells.

Cui et al. used a tough polyion complex (PIC) mixed with MWCNTs for the extrusion-based printing of a scaffold for bone repair [140]. It was indicated that the addition of MWCNTs promoted the osteogenesis of embedded mesenchymal stem cells, resulting in a mineralized matrix formation and osteogenesis-related gene upregulation. Moreover, the scaffold had remarkable mechanical properties of the tensile strength (1.69 ± 0.11 MPa), Young’s modulus (4.47 ± 0.5 MPa), and a compressive modulus (0.445 ± 0.029 MPa) and could be bent and fully recover its shape. It was used in an in vivo experiment, where it was implanted for osteogenic repair in a rat’s cranial bone. It acted as a template for new bone tissue, which had ingrown in the pores of the scaffold. The promotion of new bone formation was significantly more effective with PIC/MWCNTs than PIC alone, which further indicates the MWCNTs’ stimulatory effect.

Wang et al. [141] blended two different concentrations of MWCNTs (1% and 3 wt.%) with PCL and printed (with RegenHu Discovery bioprinter) 3D scaffold with a slice thickness of 220 µm, filament diameter of 330 µm, and pores of around 200 µm. The addition of MWCNTs slightly increased the compressive modulus, however, it vastly increased (more than two-and-a-half fold over two weeks) cell proliferation and exerted strong protein adhesion properties.

Huang et al. produced a 3D hierarchical scaffold by the screw-assisted extrusion 3D printing composed of a PCL/HA/MWCNTs composite. MWCNTs were highly aligned, surrounded by HA, which mimicked native bone structure. It was tested that MWCNTs promoted the early stage of osteogenesis differentiation of mesenchymal stem cells, while HA promoted the later stage of this process. The supplementation of PCL with MWCNTs and HA enhanced the compressive modulus from 55 to 85 MPa [142].

## 9. Discussion

Increasing demand for organs, limitations of currently existing treatment techniques, and shortage of suitable transplants have led to great advances in tissue engineering and regenerative medicine, specifically in the field of scaffold design. In terms of bone repair, two major techniques are used as a standard of treatment—autologous bone grafting and titanium implants. Both approaches suffer from patient morbidity; bone grafting is a technique with good outcomes, however, the amount of bone that can be grafted is limited [143]. Titanium implants, however, pose a risk of infection or rejection and may cause stress shielding which leads to embrittlement of the native bone [50]. In terms of articular cartilage repair, there is no effective gold standard method of treatment. Existing experimental methods rely on burdensome arthroscopic interventions combined with an influx of bone marrow, injections of stem cells, or grafting of different connective tissues. Those techniques are still in the experimental phases, with mixed outcomes and scarce long-term follow-up studies.

This led to the idea of external scaffolds, which may mimic the intrinsic complex ultrastructure of these tissues and serve as a direct replacement, which will be gradually replaced by the native tissue produced in situ by the residual cells. To achieve this, proper biocompatible materials should be developed with techniques to properly arrange the scaffold into a functional structure. Many hydrogel polymers have been tested, because of their biocompatibility and provision of aqueous environment needed to house the cells [81,92,144]. However, it was found that alone, they are too brittle to bear the load applied on either bone or cartilage. This led to the creation of composite materials where the hydrogel was reinforced with more robust molecules, such as PCL, native collagens or naturally occurring hydroxyapatite and tricalcium phosphate in terms of bone tissue. Such scaffolds were able to achieve physical characteristics comparable to the native tissues [104,145]. Due to the properties of CNTs, they were also tested for the scaffolds due to the following reasons: (1) their superior mechanical properties, with elastic moduli and tensile strength, far better than the other materials used [146]; (2) CNT size, shape, surface roughness, and surface area structurally mimics that of collagen fibers, providing a 3D network to support and guide cell proliferation, differentiation, and communication, which is not the case for the polymeric fibers, such as PCL [91,140]; (3) CNTs’ ability to interact with and adsorb extracellular proteins allows enhanced cell interaction and scaffold biocompatibility [110]; (4) CNTs offer increased cell support, which is important for angiogenesis and vascularization [147].

Nevertheless, the major problem at this stage of scaffold production is to properly arrange the fibers to, at least, obtain proper porosity, not to mention the hierarchical ultrastructure of the tissue, which is unattainable with the conventional techniques. Porosity should be high enough to provide the space for the cells and allow for the diffusion of metabolites, but the more porous the material is, the more brittle it becomes. Many conventional approaches were carried out to achieve this, e.g., using homogenous ice crystals as a mold for the polymers, which are later removed, leaving the monodisperse pores. However, the 3D bioprinting technique emerged, which is showing itself to be able to overcome the ultrastructural limitations with which scientists have been struggling to date [87]. Nowadays, 3D bioprinters offer a printing resolution of 5 µm (3DDiscovery™ Evolution, RegenHU) with several printheads and with few different printing techniques working in parallel, such as electrospinning. Nevertheless, to our knowledge, no produced scaffold exactly mimics the hierarchical ultrastructure of either bone or cartilage. The most important aspect of such scaffolds, besides the lack of toxicity, is to provide sufficient mechanical durability until the tissue regenerates. Therefore, if a scaffold possesses sufficient porosity to house the cells and sufficient mechanical properties, it seems that the fidelity in the ultrastructure of bone and cartilage can be omitted in the design of a scaffold. These ultrastructural features, such as distinct layers in cartilage (Figure 1), or a concentrated Haversian system in bone, are not necessarily required to be designed in the scaffold. Optimally, the scaffold would undergo biodegradation, at a rate similar to the ECM rebuilding, therefore these ultrastructural motifs could be remodeled by the residual cells. However, with the current state-of-the-art, it is possible to fabricate a CNT reinforced scaffold with mechanical properties similar to the native tissue, providing a microenvironment to house cells that is directly augmented by the incorporation of CNTs [140]. The scaffolds are shown to be effective in short-term in vivo studies in small animals. The current limitation in the field of 3D bioprinting is the lack of long-term research of produced scaffolds in vivo, especially in humans. Therefore, we do not have information on whether these scaffolds are sufficient to be translated to the market and used publicly. This is mostly attributed not to technical constraints because the bioprinting process itself is standardized and controlled by software, but to a high level of innovation and possible regulatory constraints preventing carrying out of such studies [147].

## Figures and Tables

**Table 1:** The table below summarizes the features of carbon nanotubes and their influence on cytotoxicity.

Aspect.	Condition	Result	116	Reference
Dose	40 μg of SWCNTs aspirated by mouse	Low toxicity	Dose probable to be encountered occupationally	[71]
Method of administration	Intratracheal instillation and inhalation	Alveolar destruction and inflammatory response upon instillation and no inflammatory cells and thickening of the alveolar wall upon inhalation	High doses of MWCNTs used	[73]
Length	5 um vs. 0.7 um MWCNTs injected peritoneally	Mesothelioma formation with long MWCNTs and no mesothelioma with short MWCNTs	-	[76]
Diameter	Macrophage viability upon exposure with <40 nm MWCNTs and 15–40 nm MWCNTs in diameter	No effects on viability with <40 nm MWCNTs and mild toxicity with 15-40 nm MWCNTs	-	[77]
Aggregation	Intratracheal instillation of aggregated and highly dispersed SWCNTs in 1% Pluronic F 108NF to mice	Lung inflammation was induced by aggregated SWCNTs in PBS, while highly dispersed SWCNTs do not cause any inflammation or fibrosis	Very high dose (40 mg) of SWCNTs was used	[79]
Purity	Cytotoxicity of MWCNTs with and without residual iron catalyst on murine alveolar macrophages	Toxic effects exerted only after treatment with unpurified MWCNTs	-	[81]
Surface functionalization	Unmodified and carboxyl modified MWCNTs instilled in C57BL/6 mouse lungs	Carboxyl functionalization reduces inflammation and lung pathologies	Dispersion status was not affecting the results, since both samples were well dispersed with surfactant	[78]
Method of detection	Toxicity of SWCNTs was tested on A549 cell line with Coomassie Blue, Alamar Blue^TM^, Neutral Red, MTT and WST-1	Employment of different method yielded various results	-	[83]
